# The Paradox of Time in Dynamic Causal Systems

**DOI:** 10.3390/e24070863

**Published:** 2022-06-23

**Authors:** Bob Rehder, Zachary J. Davis, Neil Bramley

**Affiliations:** 1 Department of Psychology, New York University, 6 Washington Place, New York, NY 10003, USA; zachdavis99@gmail.com; 2 Psychology Department, The University of Edinburgh, Edinburgh EH8 9JZ, UK; neil.bramley@ed.ac.uk

**Keywords:** causal inference, causal graphs, dynamic systems, causal learning, time, continuous, event cognition, interventions

## Abstract

Recent work has shown that people use temporal information including order, delay, and variability to infer causality between events. In this study, we build on this work by investigating the role of time in dynamic systems, where causes take continuous values and also continually influence their effects. Recent studies of learning in these systems explored short interactions in a setting with rapidly evolving dynamics and modeled people as relying on simpler, resource-limited strategies to grapple with the stream of information. A natural question that arises from such an account is whether interacting with systems that unfold more slowly might reduce the systematic errors that result from these strategies. Paradoxically, we find that slowing the task indeed reduced the frequency of one type of error, albeit at the cost of increasing the overall error rate. To explain these results we posit that human learners analyze continuous dynamics into discrete events and use the observed relationships between events to draw conclusions about causal structure. We formalize this intuition in terms of a novel *Causal Event Abstraction* model and show that this model indeed captures the observed pattern of errors. We comment on the implications these results have for causal cognition.

## 1. Introduction

Learning about causal structure is central to higher level cognition because it allows people to predict the future, select beneficial actions, and make sense of the past. The study of how people learn causal structure has historically focused on simple scenarios involving the presence or absence of binary variables (e.g., did a patient take a drug, and did they feel better?). This has taught us much about how people use causal structure for a host of decisions (e.g., [2,3,4,5]). However, this focus on simple stimuli obscures other important questions, such as how we incorporate continuous covariation and temporal information into our causal judgments.

Time is central to our notions of causality [6], making it unsurprising that temporal contiguity is one of the strongest psychological cues to causality [7]. Sophisticated expectations about delays between events shape causal judgments [8,9], interventions [10], and goal directed actions [11]. People also judge that highly variable delays are less causal [12] and use variability as a cue for structure in the absence of order or covariational cues [10].

Prior work on the role of time in causality has focused on delay distributions, i.e., the time that it takes for one event to influence another, where events are largely treated as punctate rather than extended in time. In this project we instead study a fully continuous setting in which continuous valued causes continually affect *rates of change* of their effects, introducing a different set of representational challenges. Rather than reasoning directly about rates of occurrence of events or delay distributions between events, people must reason from unfolding timeseries data.

How might varying the speed at which a continuous system evolves affect what people learn about it? Extrapolating from the literature on events cited above, one might expect that a more slowly evolving system would make learners less likely to infer the presence of causal linkages between variables. Yet a system that unfolds more slowly may have advantages as well. In the setting originally explored by [1], people were well described with a *Local Computations* (LC) model, which characterized them as focusing on establishing the relationship between pairs of variables independently, that is, rather than controlling for other variables, as one would if one considered the full space of possible structural models. The key support for the LC model came from a particular characteristic error. Participants frequently inferred direct connections between variables that were indirectly related (e.g., in the network X→Y→Z concluding incorrectly that additionally X→Z). This error was first observed in studies with binary variables observed at discrete time points [13,14]. One potential explanation of these errors in [1] is that participants failed to notice the relative time delays among the variables. In network X→Y→Z, the mediated influence of *X* on *Z* will be delayed in time compared to the direct influences of *X* on *Y* and of *Y* on *Z*. A learner who fails to notice these temporal differences will incorrectly conclude that X→Z. This hypothesis predicts that increasing the saliency of these time delay differences by slowing the system will reduce instances of these errors.

We also aim to understand how people learn causal structure from a continuous flow of information by comparing different formal accounts of how people represent continuous information and use it to infer causal relationships. Firstly, we follow [1] in describing people as computing likelihoods on the basis of the continuous dynamics directly—either considering all hypotheses in parallel (normative model), or focusing separately on individual edges (Local Computations variant). Secondly, we introduce a new account of how people might handle continuous information in time—the *Causal Event Abstraction* (CEA) model—that characterizes people as segmenting the continuous stream into discrete events, and using those to infer causal structure.

In summary, we ask two questions. Firstly, does slowing the dynamics of the system reduce the systematic errors that have been previously observed? We do find the expected reduction in those errors but at the cost of accuracy on other types of causal links. Secondly, how do people represent continuous information in dynamic systems? We find that a model describing people as segmenting continuous information into discrete events captures people’s behavior across conditions.

### 1.1. Ornstein–Uhlenbeck Networks

The stimuli in our task were generated using a new approach for simulating continuous causal systems first proposed in [15]. See [1] for a full explication of the generative process, but briefly Ornstein–Uhlenbeck (OU) networks represent causality with autoregressive processes that move towards a basin point as a function of time [16]. Importantly, however, when one variable is causally influenced by another (as defined by the causal structure of the OU network), this is modelled by making the effect’s basin point nonstationary, following some function of the state of its cause(s). Specifically, we stipulate that the basin point is the sum of the causal influences exerted by each of the effect’s causal parents. Formally, the change in a variable $v_{i}$ following time *t*, $\Delta v_{i}^{t}$, is given by

(1) $$P\left(\Delta v_{i}^{t}|v^{t},\omega ,\sigma ,\theta_{•i}\right)=\omega \left[[\sum_{j}\theta_{ji}\cdot v_{j}^{t}]-v_{i}^{t}\right]+N\left(0,\sigma \right)$$

where $v_{i}^{t}$ is the value of variable *i* at time *t*, $\theta_{ji}$ is the causal influence of variable *j* on variable *i*, $\sum_{j}\theta_{ji}v_{j}^{t}$ (the sum of $v_{i}$’s causal parents, each multiplied by its corresponding $\theta_{ji}$) is the basin to which $v_{i}$ is attracted, and σ is the endogenous noise of each variable. ω (also known as the “spring rigidity” of the system) is the rate at which $v_{i}$ reverts to its basin. For example, ω=0.10 means that the variable’s expected value will move 10% of the way toward the basin.

We now consider a number of alternative hypotheses regarding how OU networks are learned.

### 1.2. An Optimal Learner

The normative account of learning of a causal graph in an OU networks involves inverting the above generative model. (Note that although we specify normative learning in light of observed data and the interventions on the causal system made by a learner, we do not specify what interventions a learner should perform to maximize learning). Assuming an initially-uniform prior, the inferred causal structure is the one most likely to produce the changes in all variables at all time points, taking into account the learner’s interventions. Consider a hypothesis space *G* in which a learner’s task to estimate the likelihood of discrete causal hypotheses, ones where the θ associated with every potential causal relationship has been trichotomized into one of three states: positive, inverse (negative), or zero. For a system with three variables, *G* would contain 729 distinct causal hypotheses. (In this work, we exclude the possibility of self-cycles in which a variable is causally influenced by itself. That is, $\theta_{ii}=0$ for all *i*).

The likelihood of observing the change in variable $v_{i}$ at *t* given graph *g* is therefore,

(2) $$P\left(\Delta v_{i}^{t}|g,\omega ,\sigma ,\iota_{i}^{t}\right)=\int_{\theta_{•i}}P\left(\Delta v_{i}^{t}|v^{t},\omega ,\sigma ,\theta_{•i},\iota_{i}^{t}\right)P\left(\theta_{•i}|g\right)P\left(g\right)d\theta_{•i}$$

where $\int_{\theta_{•i}}$ is a multiple integral over each of $v_{i}$’s incoming causal strengths, $\theta_{•i}$. $P(\theta_{•i}|g)$ represents the priors over $\theta_{•i}$ corresponding to hypothesis *g*. For example, for a graph *g* that includes a positive X→Y causal relationship, $P(\theta_{XY}|g)=0$ for all $\theta_{XY}\leq 0$ but otherwise represents the learner’s priors over the strength of a positive causal relationship when $\theta_{XY}>0$.

$\iota_{i}^{t}$ is an indicator variable that is true if $v_{i}$ is intervened on at *t* and false otherwise. We accommodate interventions by the standard notion of graph surgery [17]. Thus, if $v_{i}$ is manipulated at time *t*, the likelihood of the observed $\Delta v_{i}^{t}$ is 1 (i.e., is independent of $v_{i}$’s current value or the value of its causes). Otherwise, it is given by Equation (1). That is,

(3) $$P\left(\Delta v_{i}^{t}|v^{t},\omega ,\sigma ,\theta_{•i},\iota_{i}^{t}\right)=\left\{\begin{array}{cc} 1 & \iota_{i}^{t}\;\mathrm{True}\\ N(\omega \left(\sum_{j}\theta_{ji}v_{j}^{t}-v_{i}^{t}\right),\sigma ) & \iota_{i}^{t}\;\mathrm{False} \end{array}\right.$$

The likelihood of all observed variables at all time points, taking into account potential uncertainty regarding ω and σ, is,

(4) $$P\left(v|g,\iota \right)=\prod_{i=1}^{N}\prod_{t=1}^{T-1}\int_{\omega }\int_{\sigma }P\left(\Delta v_{i}^{t}|v^{t},g,\omega ,\sigma ,\iota_{i}^{t}\right)P\left(\omega \right)P\left(\sigma \right)d\omega d\sigma$$

P(ω) and P(σ) represent the learner’s priors over ω and σ. See [1] for additional details and explanation.

#### Simulations of an Optimal Learner

**Figure 1:**
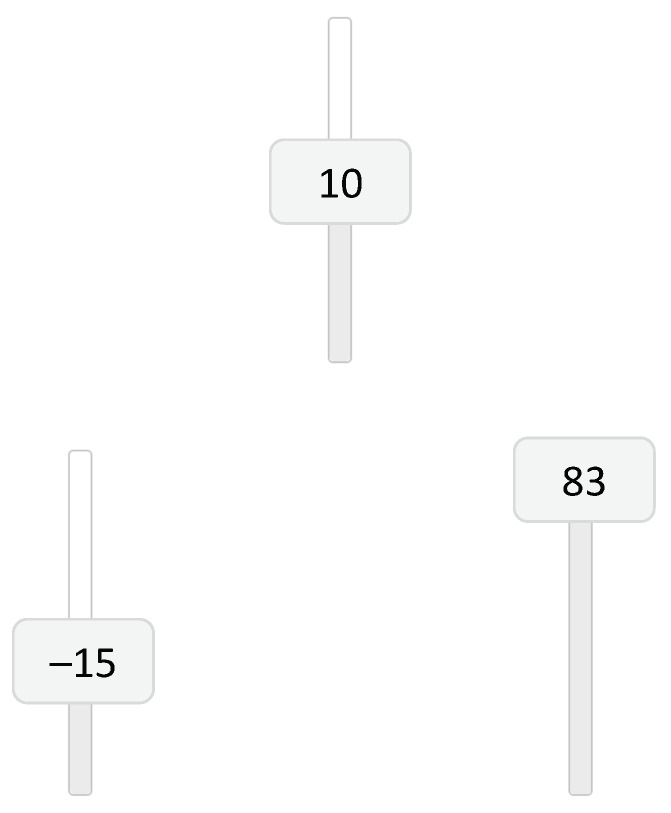
An OU system with three variables displayed as “sliders” on a computer screen. The values of the sliders take on values from −100 to 100 and are updated continuously as a function of the input they receive from their causal parents and system noise σ.

We now present simulations of an optimal learner to identify some key factors that determine its success at learning a causal network. Several assumptions were made to make these simulations relevant to the experiment that appears at the end of this paper. In that experiment, the variables of the OU system are presented as sliders that take on a value between −100 and 100 (see Figure 1 for an example). Human learners are asked to identify the causal structure that relates these variables.

**Figure 2:**
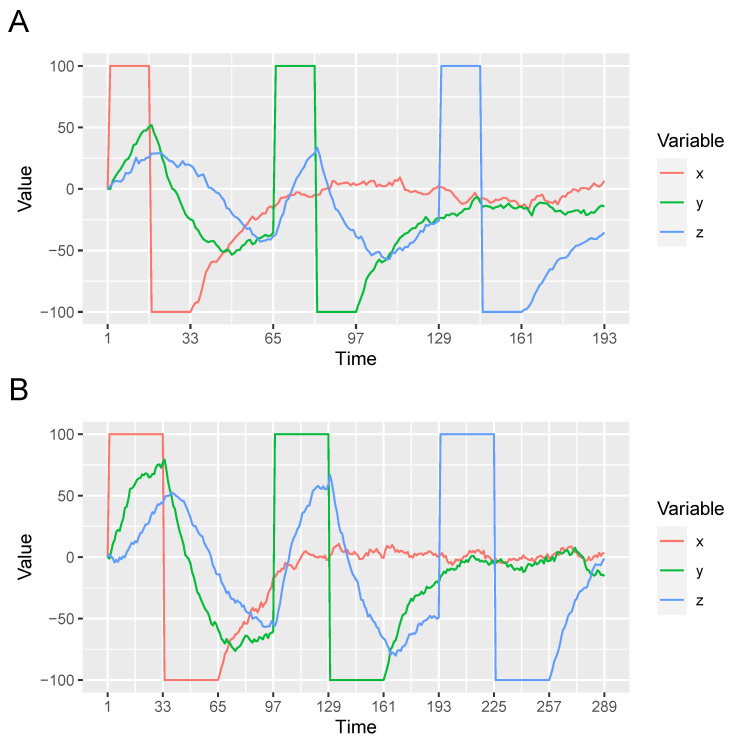
Examples of manipulating an OU network with three variables that form a causal chain X→Y→Z. In each panel *X* is first manipulated, followed by *Y* and then *Z*. Each manipulation consists holding the variable at 100 and then −100. Panels (**A**,**B**) present manipulations that last 32 and 64 time units, respectively. Interventions were separated by 32 time units, allowing the variables to return to a baseline value near 0. The resulting changes in in *X*, *Y*, and *Z* reflect the X→Y→Z causal relationships. $\theta_{XY}=\theta_{YZ}=1$, ω = 0.05, and σ = 2.

First, because learners will be allowed to manipulate the variables of the OU network, our theoretical analysis will assume the presence of manipulations qualitatively similar to those observed in [1]. In particular, we assume that each variable is manipulated by first setting it to one extreme value (100) and then the other (–100) during each learning trial. Figure 2 shows examples of the variable manipulations that were presented to the optimal learner.

Second, the upcoming experiment will present subjects with four instructional videos. These will present examples of OU systems with values of ω and σ, and possible values of θ (−1, 0, or 1), that are the same as those of OU systems they are subsequently asked to learn. Thus, for simplicity the simulations were derived assuming that learners extract from the videos those values of ω and σ and the possible values of the θs.

Third, without modification the normative model is powerful enough to almost perfectly identify the correct hypothesis given the amount of time subjects are allowed to examine how the OU network evolves over time. We think that such extreme performance is psychologically unrealistic because human learners presumably experience simple resource limitations (e.g., lapses of attention). Thus, in presenting the simulation results we will pass the normative model’s posterior probabilities through a softmax function.

(5) $$P\left(g|v,\iota \right)=\frac{P{\left(v|g,\iota \right)}^{-\tau }}{\sum_{k}P{\left(v|g,\iota \right)}^{-\tau }}$$

Values of τ<1 yield a posterior distribution over *G* that is less “sharp”, that is, one that favors the true hypothesis less decisively than it would otherwise. In the simulations below τ=40.

Note that it is straightforward to go from a posterior distribution over *G* to the posterior probability of a positive, negative, or zero causal relationship from one variable to another via Bayesian model averaging. Define $G_{l}$ as the subset of graphs that includes a particular causal link *l* (e.g., a positive X→Y causal relationship). Then, the posterior probability of *l* is simply,

(6) $$P\left(l|v,\iota \right)=\sum_{g\in G_{l}}P\left(g|v,\iota \right)$$

Our simulations focus on the chain network X→Y→Z because it is an example of a causal system that is susceptible to the local computations error described earlier (i.e., incorrectly inferring that *X* and *Z* are directly rather than indirectly causally related). The normative model’s ability to learn X→Y→Z is examined as a function two properties, properties that turn out to discriminate an optimal learner from the two alternative models described later. The first is the OU network’s spring rigidity ω. The second is a property of the variable manipulations that we refer to as *intervention duration*. Intervention duration is the amount of time that a variable is manipulated to both extreme values (100 or –100). Whereas in Figure 2A the manipulation of each variable lasts 32 time steps, in Figure 2B they last 64 time steps.

**Figure 3:**
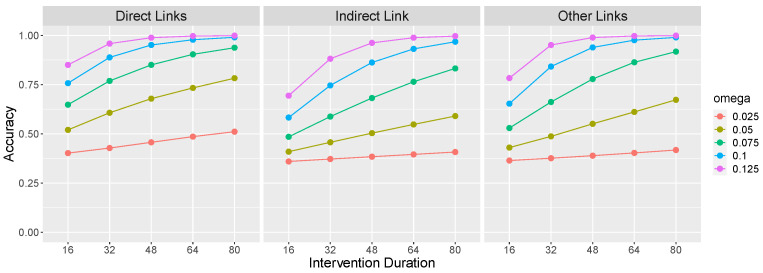
Accuracy for an optimal learner learning the causal graph X→Y→Z as a function of ω and intervention duration. The first panel presents accuracy at correctly identifying the presence of the X→Y and Y→Z causal relationships. The second panel presents accuracy at correctly identifying the absence of an X→Z causal relationship. The third panel presents accuracy at correctly identifying the absence of the remaining potential causal relationships (Y→X, Z→Y, Z→X). $\theta_{XY}=\theta_{YZ}=1$, σ = 5 and τ = 40. Results are averaged over 1000 simulations of each parameter combination.

Figure 3 presents learning accuracy on the X→Y→Z causal network as a function of ω and intervention duration. Direct links (left panel of Figure 3) refers to the average accuracy on the causal links that make up the causal chain, namely, X→Y and Y→Z. Accuracy on these links consists of correctly identifying the presence of a link between these pairs of variables. The indirect link (middle panel) refers to a potential X→Z link. Because there is no such link in the X→Y→Z causal chain, accuracy consists of correctly identifying the *absence* of such a causal relationship. Other links (right panel) refers to other potential causal relations between the variables (i.e., Y→X, Z→Y, Z→X), and again accuracy consists of correctly identifying the absence of those relations.

Figure 3 confirms that an important factor determining the learnability of an OU network’s causal relations is its rigidity ω: Causal links are more easily identified when an effect variable exhibits a larger change in value (due to a larger ω) in response to a change in value of its cause. This is so because a large change is less likely to be due to system noise. Of course, this result generalizes findings reviewed above that temporal contiguity between events promotes the identification of causal relations to continuous variables that react more quickly to causal interventions.

Figure 3 also reveals that longer interventions also aid learning. This is so for a reason that is analogous to the effect of rigidity: A longer intervention allows more time for a change to become apparent against a background of system noise.

**Figure 4:**
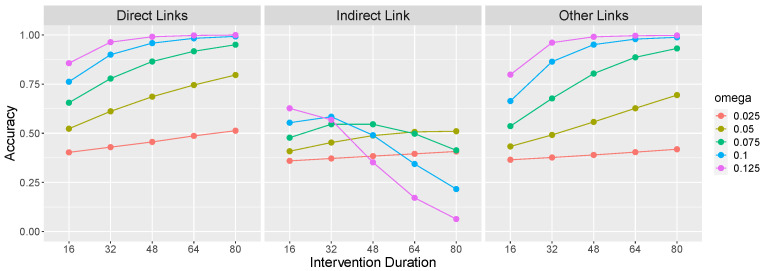
Accuracy of the Local Computations (LC) model under the same parameterization as Figure 3. Results are averaged over 1000 simulations of each parameter combination.

**Figure 5:**
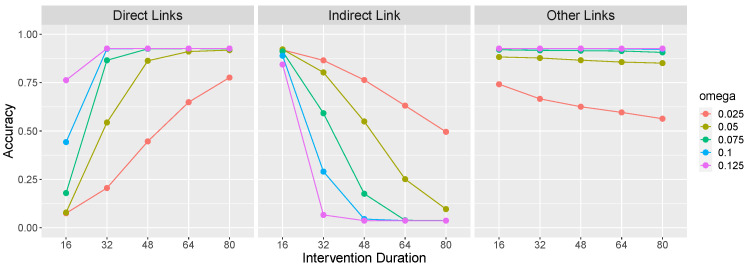
Accuracy of the Causal Event Abstraction (CEA) model under the same parameterization as Figure 3 and Figure 4. CEA’s threshold parameter was 50 and its guessing parameter was 0.10. Results are averaged over 1000 simulations of each parameter combination.

Note that another important factor that influences learning in OU systems (one not shown in Figure 3) is the *range* of the intervention, that is, the absolute magnitude of the change that the manipulated variable undergoes. Whereas in Figure 3 the variables are manipulated to their extreme values of 100 and −100, less extreme manipulations will result in degraded learning. In our Supplementary Materials (https://osf.io/rfx2q) we present simulations that vary intervention range while holding intervention duration constant that show results analogous to those in Figure 3, Figure 4 and Figure 5. We will also evaluate the effect of both intervention duration and range when presenting the results of the upcoming experiment.

### 1.3. The Local Computations Model

We compare an optimal learner to the Local Computations (LC) model. As mentioned, LC has been advocated as a general-purpose account of causal learning behavior [13,18]. Applied to an OU network, the LC model entails deciding, for each potential causal relationship considered in isolation, whether the observed values of those two variables implies a positive, inverted (negative), or zero causal relation.

LC can be formalized by rewriting Equation (3) in the case that $\iota_{i}^{t}$ is false with,

(7) $$P\left(\Delta v_{i}^{t}|v^{t},\omega ,\sigma ,\theta_{•i}\right)=\sum_{j,j\neq i}^{N}N\left(\omega \left(\theta_{ji}v_{j}^{t}-v_{i}^{t}\right),\sigma \right)$$

Whereas Equation (3) computes the probability of observing $\Delta v_{i}^{t}$ by considering the simultaneous influences of all of $v_{i}$’s causal parents, Equation (7) does so by considering each parent in isolation, failing to control for the fact that $\Delta v_{i}^{t}$ might partly be due to one of the other causal parents. For example, given an OU network with three variables *X*, *Y*, and *Z*, the likelihood of a change in, say, *Z*, $\Delta v_{Z}^{t}$, is computed by computing the likelihood of the $\Delta v_{Z}^{t}$ given *X* ignoring *Y*, the likelihood of the $\Delta v_{Z}^{t}$ given *Y* ignoring *X*, and summing the two. LC-based models have been proposed as accounts of how people build causal models in a resource-efficient way [13,19].

#### Simulations of an LC Learner

Figure 4 shows the performance of the LC model on the causal graph X→Y→Z as a function of the same parameters as in Figure 3. Like the normative model, LC’s performance generally improves as the rigidity ω and intervention duration increase. However, the middle panel reveals that these same factors result in LC becomes increasingly vulnerable to committing local computation errors (i.e., incorrectly inferring X→Z). Indeed, a rigid OU system with ω = 0.125 and interventions of length 80 will almost certainly be perceived as including a X→Z causal relationship in addition to X→Y and Y→Z. This is so because a long intervention on *X* combined with a large ω results in a large and rapid change to *Z*, which is easily mistaken as evidence for X→Z.

### 1.4. The Causal Event Abstraction Model

Whereas the normative learning model and the LC model both compute likelihoods associated with the observed data, the *Causal Event Abstraction* (CEA) model posits that people use a simple heuristic to identify causal relations. In particular, it assumes that, while one variable of an OU system is being manipulated, people track the changes that occur to the system’s other variables. Should a change to a variable during that intervention be sufficiently large, it is recorded as a change ‘event’ providing evidence for a causal relationship from the manipulated variable to the changed one.

**Figure 6:**
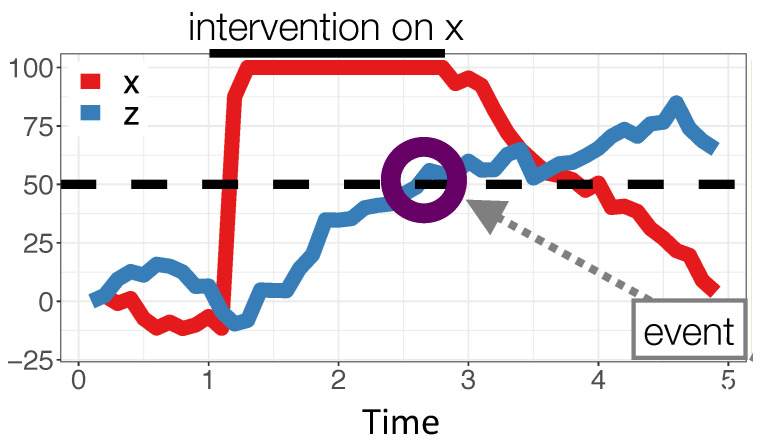
Illustration of the CEA model. During the learner’s manipulation of *X*, which takes place during seconds 1–3, *Z* crosses threshold (here shown as 50).

CEA’s main parameter is the *threshold* value that the absolute value of the purported effect variable must exceed during an intervention to be classified as undergoing a change. In the simulations below, the threshold is 50 and so a change event is recorded if the variable goes above 50 or below −50. For example, Figure 6 shows variable *Z* changing in response to a manipulation on *X*. Because *Z* exceeds the threshold (dashed line in Figure 6) a change event would be recorded as evidence for a causal relation between *X* and *Z* (To only register events when a threshold is *crossed*, CEA excludes all cases where a potential end variable is above threshold before the intervention begins). For all timepoints during the intervention that the variable exceeds the threshold, CEA compares the signs of it and the manipulated variable and records evidence for a regular (positive) causal link if on average the signs match and an inverse (negative) one otherwise. For example, after *Z* exceeds the threshold in Figure 6, the sign of both it and *X* are positive so the change event would be recorded as evidence for a positive X→Z relationship. For variables that did not change during the intervention, no evidence of a causal link between it and the manipulated variable is recorded.

The probability of a causal relationship (say, a positive X→Z relationship) is then computed by CEA by dividing the number of positive changes to *Z* induced by the manipulation of *X* divided by the number of times that *X* was manipulated. This calculation is also moderated by a guessing parameter (0.10 in the simulations) that corresponded to the probability of responding counter to the predictions of the events model. Note that the CEA model is insensitive to temporal delays in that it only depends on whether a variable exceeds the threshold, not how quickly. It only infers a causal relationship from a variable if that variable has been manipulated at least once.

#### Simulations of a CEA Learner

Figure 5 show the performance of the CEA model on the causal graph X→Y→Z as a function of both spring rigidity (ω) and intervention duration. As in the previous models, CEA’s success at identifying the X→Y and Y→Z causal relations (left side of Figure 5) generally increases as ω increases. Unlike the previous models however, accuracy on the relations also increases sharply as the duration of the interventions increase. This is so because short interventions will not allow sufficient time for the effect variables to cross the threshold.

In addition, the middle panel of Figure 5 reveals that CEA is also vulnerable to committing local computation errors (incorrectly inferring X→Z), just as LC is. This panel reveals that both increasing ω and intervention duration result greater local computation errors. This is so because both of these factors increase the probability that *Z* will cross the threshold in response to a manipulation of *X*.

### 1.5. Summary of Learning Models

The following experiment tests these model predictions by explicitly manipulating the rigidity parameter ω, varying it between the values of 0.05, representing a more flexible system that responds more slowly to changes in inputs, and 0.10, representing a more rigid system that responds more quickly. We also analyze how learning success varies with the duration and range of the interventions that learners choose to make.

**Figure 7:**
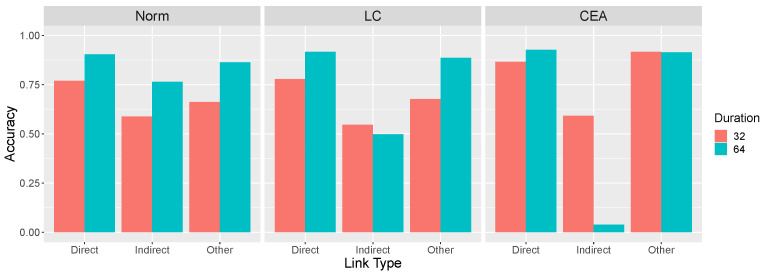
Predictions of the three models for three causal link types for intervention duration of 64.

Figure 7 summarizes the predictions shown in Figure 3, Figure 4 and Figure 5 for ω values of 0.05 and 0.10 and an intervention duration of 64. Figure 7 reveals that the LC and CEA models capture what we have referred to as the paradox of time in learning causal systems. Generally, these models predict that the correct identification of both the presence and absence of causal relationships is promoted when a learner’s interventions result in a system undergoing more rapid changes due to a larger ω. However, more rapid changes also makes it more likely that these models will incorrectly conclude that two variables that are indirectly causally related (*X* and *Z* in X→Y→Z) have a direct causal relation between them. We ask whether human learners also exhibit this pattern. We also predict that longer and more extreme interventions will have an effect that is analogous to rigidity, namely, better performance overall but more local computation errors. Finally, we fit all three models to the learning results to determine which model provides the best quantitative account of the data.

## 2. Materials and Methods

### 2.1. Participants

107 participants were recruited from Amazon Mechanical Turk using psiTurk [20]. They were paid a base payment of $3 plus performance related bonuses (M = $0.97, SD = $0.46) and the task took 32.6 minutes (SD = 18.3). Participants were randomly assigned to either the rigid or the flexible condition. Those who made a causal judgment before intervening on any slider on over 90% of trials were excluded, leaving 87 participants (29 female, 58 male; age M = 37.6, SD = 11.8). The results presented below are based on 42 and 45 participants in the flexible and rigid conditions, respectively.

### 2.2. Materials

**Figure 8:**
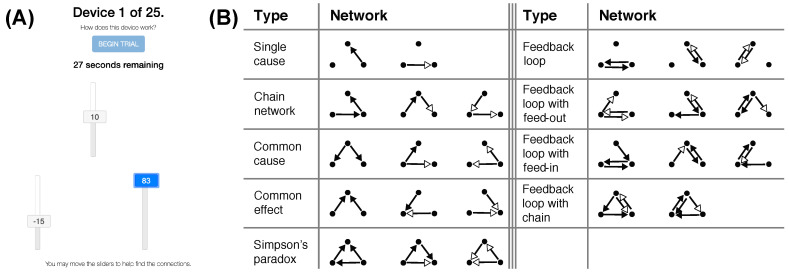
Stimuli. (**A**) Task environment. Sliders turn blue when intervened on. (**B**) All tested causal graphs, presented in random order. Black arrowheads denote regular connections, white arrowheads denote inverse connections.

Participants interacted with a number of causal devices represented by three vertical sliders that moved on their own according to the hidden causal structure and OU process, but could also be intervened on, by clicking and dragging to set their levels, overriding their normal causes (see Figure 8A) (See zach-davis.github.io for a demo). The sliders were constrained to be between −100 and 100, and the buttons on the slider presented a rounded integer value in addition to moving up and down. A timer at the top of the page counted down from 45 s at 1 s increments, and at the bottom of the page were six additional sliders (one for each potential causal relation). Responses could be one of three options: ‘Inverted’, ‘None’, or ‘Regular’, corresponding to θ<0, no relationship (θ=0), and θ>0, respectively. Participants were pretrained on these terms in the instructions.

### 2.3. Stimuli and Design

Participants were tested on 25 causal graphs (see Figure 8B) that were roughly balanced across a number of factors, such as the number of inverted and regular links and the number of links between each variable. The graphs were presented in random order for a total of 25 trials. The OU parameters used during training and the test were σ=5 and θ=[1,0,−1] for regular, none, or inverse connections, respectively. The sliders were updated with the OU system’s next set of variable values every 100 ms.

**Figure 9:**
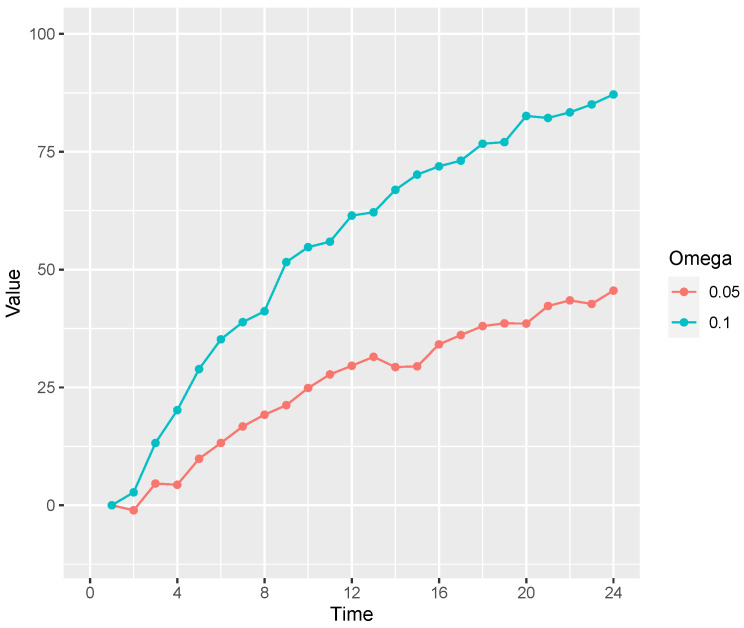
An OU variable’s rate of change toward a basin of 100 for two values of ω. Stimuli were generated with a small amount of noise (σ=2).

Participants were randomly assigned to one of two conditions in which the rigidity ω parameter was either 0.05 (“flexible”) or 0.10 (“rigid” condition). Recall that ω sets the rate at which the process asymptotes: When ω=0.05 (0.10) a variables move 5% (10%) of the way toward its current basin (see Figure 9).

### 2.4. Procedure

Participants first completed an interactive instruction section that used a sequence of videos to explain the nature and goals of the task, how to intervene, as well as the trial duration. They were instructed that, for a randomly selected trial, they would receive a bonus of $0.25 for each correct causal link judgment (out of ‘no link’, ‘regular’ and ‘inverse’ for each of the 6 directed links). Importantly, this bonus scheme was demonstrated with a hypothetical participant who observed a chain network and correctly identified the two existing causal links but incorrectly added an additional direct link between the indirect effects. Participants were told that this participant received a reward of $1.25 for the correct responses but missed out on an additional $0.25 for marking the direct connection between indirect effects. Participants could not proceed to the task until they correctly answered five comprehension check questions probing if they knew the duration of each trial, the difference between a regular and inverted connection, that there can be more than one connection per network, and that they would have to provide a response for all six possible connections on each trial.

In the main task, participants completed 25 trials lasting 45 s each. A trial was initiated by pressing the “Start” button at the top of the page, whereupon the sliders began updating according to the OU process every 100 ms. Participants were free to click, drag, or hold any slider to any value for any amount of time, overriding its normal causal input, if any. After releasing a slider, it continued to move according to the OU process.

Participants could make (and revise) their causal judgments at any point during the trial, but could not proceed to the next trial until they had entered a judgment for all six potential causal relations. No feedback was provided. After completing the 25 trials, participants were informed of their bonus and completed a brief post-test questionnaire.

## 3. Results

Across all conditions, participants were above chance (0.33) in identifying causal links (M = 0.763, SD = 0.203), t(86)=19.80, p<0.0001. They were slightly more likely to correctly identify regular (0.869) than inverse (0.837) causal links, t(86)=3.14, p=0.002. Participants were also more likely to correctly classify causal links as the experiment progressed, as confirmed by a regression with subject-level intercept and slope for trial number (mean β=0.004), t(86)=5.24, p<0.001. Accuracy was 0.789, 0.788, 0.753, and 0.642 for OU networks with 1, 2, 3, and 4 causal links, respectively, F(3,258)=23.3, p<0.0001, indicating that learning difficulty increased with the complexity of the network.

### 3.1. Effect of Rigidity on Accuracy

**Figure 10:**
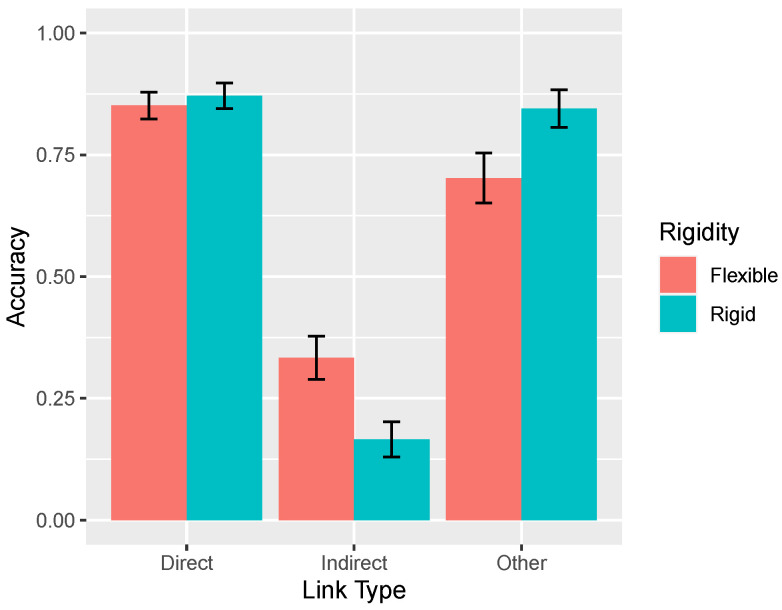
Accuracy identifying causal links by rigidity condition (ω = 0.05 or 0.10) and type of causal link. Causal links are categorized in the same manner as Figure 3, Figure 4, Figure 5 and Figure 6, namely, as direct, indirect, and other. For example, in a X→Y→Z network the direct links are X→Y and Y→Z, the indirect link is X→Z, and the other links are Y→X, Z→Y, Z→X. Accuracy on direct links means correctly identifying the presence of a causal link (and its sign) and accuracy on the remaining links means correctly identifying their absence. Error bars are standard errors of the mean.

Consistent with the theoretical analyses presented earlier, overall accuracy increased as the rigidity of the system increased, from 0.731 in the flexible (ω = 0.05) to 0.800 in the rigid (ω = 0.10) condition, an effect that was marginally significant t(86)=1.50, p= 0.137. However, the key theoretical question is how accuracy varied with type of causal link across rigidity conditions, as shown in Figure 10. In the rigid condition, accuracy was generally good, except for the very poor (indeed, below chance) performance on the indirect links. This result reflects learners’ tendency to mistakenly infer a direct causal relationship between two variables that are only indirectly related (e.g., *X* and *Z* in X→Y→Z) and replicates past findings [1]. The important result is that this pattern of errors interacted with the manipulation of ω: When the system was more flexible, accuracy decreased on the direct and other links but, paradoxically, improved on the indirect links.

These findings were supported by statistical analysis. A two-way mixed ANOVA with repeated measures on the link type factor revealed a main effect of link type F(2,170)=204.1, p<0.0001, no main effect of rigidity F<1, but an interaction, F(2,170)=12.7, p<0.0001. Accuracy on indirect links decreased as system rigidity increased, t(85)=3.17, p=0.002. In contrast, accuracy on other links increased, t(85)=2.20, p=0.030. Accuracy on the direct links also increased, although not significantly so, t<1. Note that the total number of causal links inferred per causal network in the flexible (3.51) and rigid (3.19) conditions were not significantly different, t(85)=1.50, p=0.137.

### 3.2. Effect of Interventions on Accuracy

**Figure 11:**
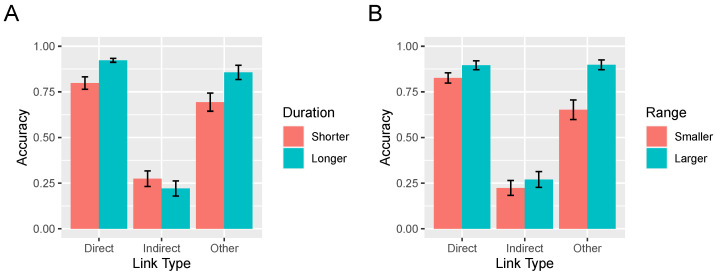
(**A**) Accuracy identifying causal links by intervention duration and type of causal link. (**B**) Accuracy identifying causal links by intervention range and type of causal link. Error bars are standard errors of the mean.

As mentioned, successful learning relies on effective interventions, that is, ones that are extended in time and involve large swings of each variable’s value. The average intervention duration did not differ between the flexible (3.86 s) and rigid (3.79 s) conditions, t<1. To assess how the duration of participants’ intervention affected their learning, we repeated the 2 × 3 analysis corresponding to Figure 10 with intervention duration added as a per-participant covariate. This analysis yielded an effect of intervention duration, F(1,83)=8.08, p=0.006, indicating that longer interventions were associated with greater accuracy, but also an interaction between duration and causal link type, F(2,166)=20.18, p<0.0001. This interaction is depicted in Figure 11A in which interventions have been dichotimized via a median split into those that are short and long. Although overall accuracy improved as the duration of interventions increased, accuracy on the indirect links was lower when interventions were longer. The explanation for this result is straightforward. For example, in the network X→Y→Z, longer interventions allow time for the value of variable *Z* to change in response to an intervention on *X*, allowing the learner to incorrectly infer the existence of a direct X→Z relationship. Separate analyses of each link type revealed that longer interventions resulted in significantly higher accuracy on direct and other links (both *p*s <0.0001) and marginally lower accuracy on the indirect links, t(85)=1.59, p=0.121. Note that the two-way interaction depicted in Figure 11A did not itself significantly interact with rigidity condition, F(2,166)=2.01, p=0.138.

The average range of interventions—defined as the minimum slider value subtracted from the maximum value during an intervention bout—was 141.4 in the rigid condition as compared to 126.5 in the flexible condition, a difference that arose because rigid condition participants were more likely to swing the variable between extremes (e.g., from 100 to −100). This difference did not reach statistical significance however, t(85)=1.59, p=0.115. To assess how intervention range affected learning, we again repeated the 2 (rigidity condition) × 3 (link type) analysis now with intervention range as a per-participant covariate. This analysis yielded an effect of range, F(1,83)=28.1, p<0.0001, indicating that interventions of a larger magnitude were associated with greater accuracy, but also an interaction between range and causal link type, F(2,166)=5.19, p=0.007. This interaction is depicted in Figure 11B in which intervention range has been dichotimized via a median split into smaller and larger. The interaction reflects the fact that the increase in accuracy brought about by increased range was lower for indirect links than the other link types. Again, this result is explicable under the assumption that larger interventions increase the likelihood that indirect causal links will be mistaken for direct ones. Separate analyses of each link type revealed that more extreme interventions resulted in significantly higher accuracy on direct and other links (both *p*s <0.0001). In contrast, accuracy on the indirect links did not vary with range, t(85)=1.09, p=0.279. The two way interaction in Figure 11B between range and link type did not itself interact with rigidity condition, F<1.

### 3.3. Modeling

To better understand participants’ judgments, we compared them to the causal structure learning models presented above. For each participant and model, the model received as input the slider values and the participant’s interventions and yielded a posterior distribution over the 729 causal graphs. As mentioned, the normative model inverts the generative model to optimally infer the structure most likely to have produced the evidence. We assumed a uniform prior over the hypothesis space. We also assumed priors over the parameters ω, θ, and σ. Because they observed four instructional videos of OU networks with those parameter values, we assume that subjects induced the true values of those parameters albeit with some uncertainty. (See our Supplementary Materials at https://osf.io/rfx2q for details). A softmax function was applied to the posterior over graphs, with a separate temperature parameter τ fit for each participant.

The Local Computations (LC) model focuses on pairs of variables rather than evaluating the evidence with respect to the full space of possible causal models (Equation (7)). In other respects the LC model is identical to the normative model. Note that [1] showed that the LC model best fit participants in a very similar task to this study’s rigid condition. Here we test the extent to which these results generalize to different time characteristics.

The Causal Event Abstraction (CEA) model describes people as abstracting continuous variables into events and using those events as cues for causality. To account for uncertainty in participant judgments, we fit not only a per participant threshold parameter but also a guessing parameter that corresponded to the probability of responding counter to the predictions of the events model.

Finally, we compare the models above to a baseline model that assumes participants have an equal probability of responding for any graph. It has no fitted parameters.

### 3.4. Modeling Results

For the normative model, the median fitted values of the softmax τ parameter was 6.15 and 6.35 in the flexible and rigid conditions, respectively, whereas for the LC model they were 5.98 and 6.81. For the CEA model, the median fitted values of the threshold and guessing parameter were 53.1 and 0.284, respectively, in the flexible condition and 64.3 and 0.107 in the rigid condition.

**Figure 12:**
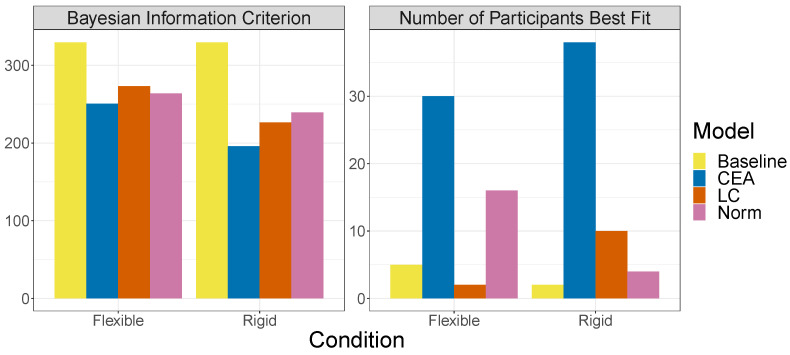
Evaluation measures for the theoretical models. Left panel: Mean BIC per participant. Right panel: Number of participants best fit by each model as measured by BIC. The normative and LC models were fit with a softmax temperature parameter per participant. The CEA model was fit with a threshold and guessing parameter per participant.

The left panel of Figure 12 shows the relative performance of the models as measured by mean Bayesian Information Criterion (BIC) per participant. Overall, CEA is the best-fitting model. This greater performance of CEA is also reflected in the number of participants best fit by each model (right panel of Figure 12). Although the CEA model fits the majority of participants in both conditions, its advantage over the other models was slightly greater in the rigid as compared to the flexible condition.

Note that the CEA models also explains one way that learners’ interventions varied across experimental conditions. For example, a good intervention for the CEA model involves holding an intervened-on variable at or near a particular value for an extended period (providing the time needed for an effect variable to cross its threshold so that an event is recorded). Although the duration and range of interventions did not vary significantly with rigidity, our Supplementary Materials (https://osf.io/rfx2q) presents the proportion of interventions that are held *at one value* over time in each experimental condition. In fact, as the time increased for a variable to cross some threshold because of lower rigidity, learners were more likely to hold the intervened-on variable at one value, a behavior consistent with a CEA learner.

### 3.5. Replication Experiment

We augment these results by reporting in our Supplementary Materials (https://osf.io/rfx2q) the results of a replication experiment that was identical except that the rate at which the computer screen was updated to include the next OU system state (100 ms in the current experiment) was set to 300 ms instead. The results were qualitatively identical, including the interactions shown in Figure 10 and Figure 11 and the general superiority of the CEA model.

## 4. Discussion

This paper investigated the impact of timing on causal learning in continuous dynamic systems. Specifically, by manipulating an OU system’s rigidity we varied the rate at which causes influence their effects. We hypothesized doing so would moderate a particular type of error previously captured by the Local Computations model—given X→Y→Z, incorrectly inferring a direct relationship between *X* and *Z*—because in a less rigid system learners would be more likely to note that the influence of *X* on *Z* was time delayed, making the possibility that this relationship was mediated by *Y* more salient. Yet, we also noted that people are generally less likely to infer a causal relationship the greater the time delay between cause and effect. In fact, we found just this paradoxical effect of time on learning: While slowing the dynamics resulted in increased accuracy for indirect effects, it also resulted in reduced accuracy on other types of causal links. That is, rather than having a uniformly positive or negative effect, changes in system timing led to a trade-off between different types of errors.

Although we could not manipulate the interventions that learners chose to make, we also predicted that both the duration and range of those interventions would have effects that were analogous to those of rigidity. In fact, we found that longer interventions were associated with better learning performance overall but at the cost of increasing the prevalence of local computation errors. Interventions of a greater range (i.e., achieved by setting intervened-on variables to more extreme values) were also associated with better overall performance. Although greater range did not numerically increase local computation errors, it did not improve performance on the indirect links as it did on the other link types. Note that the interactions between the pattern of errors and system rigidity, intervention duration, and intervention range were not predicted by the optimal learning model.

To make sense of this pattern of results, we drew on a foundational principle in cognitive psychology: that a major part of what brains do is abstract and discretize continuous inputs into quantities and concepts amenable to structured symbolic processing [21,22]. Along these lines, we explored the idea that people form a greatly simplified representation of the causal dynamics they are observing, viewing them as constituted by causal events triggered by interventions, and using this representation to drive their structure inferences.

We introduced this principle in the form of the Causal Event Abstraction (CEA) model, finding that it better captured the majority of our participants. The success of this model fits nicely with work suggesting that people naturally abstract continuous streams of information into discrete events (for review, see, [22]). That said, the CEA model in its current form is highly exploratory with plenty of room for improvement and further testing. First, CES’s current notion of a threshold is absolute in that it is defined relative to 0. This was perhaps a reasonable simplifying assumption for the OU networks tested here in which variables tended to revert to a basin of 0 in the absence of interventions. In other setting, a more realistic model would consider the change in a variable relative to its starting value. Second, CEA’s threshold is also binary: An effect variable either crosses it or not. In reality, evidence for a causal relation in human learners may be more graded in that it depends on the distance from the threshold. (We thank an anonymous reviewer for mentioning this possibility). Third, in its current form CEA only infers a direct connection between an intervened-on root variable and end variable that registers an effect, whereas people have been shown to infer structure by linking sequences of events [10]. Fourth, future studies could apply the event abstraction principle as an account of observational causal inference as well as interventional learning. Fifth, given the importance of interventions to produce events for the CEA to learn from, a future direction would be modeling the CEA’s prescriptions for how one should intervene to maximize learning. It seems probable that the a goal of producing causally-indicative event sequences would predict markedly different behaviours than the goal of generating the most normatively “invertable” continuous dynamics. Finally, the real-time setting explored here also has rich implications for issues of bounded rationality in active learning. For instance, given the potentially overwhelming complexity of real time dynamics, learners might choose interventions that generate evidence that is informative but not so complex that it cannot be used (cf. [23,24,25]).

While we manipulated the “speed” of the system dynamics here, even our supposedly slow (i.e., flexible) condition reflects what we believe is the fast end of the spectrum of the dynamics people reckon with in daily life. From economic conditions to climate patterns, many decision-relevant causal dynamics unfold orders of magnitude slower that those we probed in this experiment. It is an open question what relationship such radical clock-time shift has on the interactions between human cognition, intervention choice, event abstraction and causal learning. Recent work examining causal inference from observations spanning hours [26] and days [27] suggests people have at least as much difficulty identifying relationships and dealing with confounds and dependencies. In such settings it seems likely that processing bottlenecks are caused as much by the structure and limits of long term memory and retrieval as by limited online processing bandwidth.

Learning the relationships between continually shifting variables in real-time is as challenging as it is common. In this paper, we identified factors that modulate performance in continuous dynamic environments, and proposed a new model for causal learning inspired by people’s ability to abstract and discretize their experiences. We find support for the idea that, in these informationally rich settings, people use events triggered by their actions to infer causal structure.

## Data Availability

Empirical data for this experiment can be accessed at Supplementary Materials (https://osf.io/rfx2q).

## Supplementary Materials

The following supporting information can be downloaded at: https://osf.io/rfx2q, accessed on 2 April 2022.
